# Supplementary material for: Total Knee Arthroplasty in Patients with Primary Sjögren’s Syndrome: A Retrospective Case-Control Study Matched Patients without Rheumatic Diseases
Source: J Clin Med. 2022 Dec 15;11(24):7438. doi: 10.3390/jcm11247438 (PMC9786774; doi:10.3390/jcm11247438)
Supplement: Supplementary file 1 [file jcm-11-07438-s001.zip › jcm-1998217-supplementary.pdf]

Supplement Table S1. Characteristics of pSS patients

	Data	Rate (%)
Clinical characteristics		
Age at diagnosis(years)	55.87±7.81	-
PSS Duration(years)	7.47±4.79	-
Lung involvement (Y/N)	3/27	10.00
Renal involvement (Y/N)	2/28	6.67
Digestive involvement (Y/N)	2/28	6.67
Nervous system involvement (Y/N)	0/30	0
Hematological involvement (Y/N)	10/20	33.33
Autoimmune thyroid disease (Y/N)	1/29	3.33
Smoking (Y/N)	1/29	3.33
ESSPRI	6.38±1.71	-
Laboratory characteristics		
Anti-SSA (+) (Y/N)	13/11	54.17
Anti-SSB (+) (Y/N)	10/14	41.67
ANA (+) (Y/N)	22/5	81.48
RF (+) (Y/N)	15/12	55.56
Low C3 (Y/N)	9/18	33.33
Low C4 (Y/N)	8/19	29.63
High IgG levels (Y/N)	13/14	48.15
Treatments characteristics		
Glucocorticosteroids (Y/N)	26/4	86.67
Perioperative “stress-dose” steroids (Y/N)	22/8	73.33
Hydroxychloroquine (Y/N)	21/9	70.00
Total glucosides of paeony (Y/N)	19/11	63.33
bDMARDs (Y/N)	2/28	6.67

tsDMARDs (Y/N)	0/30	0.00

Abbreviations: ESSPRI= EULAR Sjogren's Syndrome Patient Reported Index; Low C3 =C3<0.73g/L; Low C4 =C4< 0.1g/L; High IgG levels= IgG ≥17.00 g/L; bDMARDs= biological disease modifying anti-rheumatic drugs; tsDMARDs= target synthetic disease modifying anti-rheumatic drugs
